# Regulation of erythroid differentiation in K562 cells by the EPAS1-IRS2 axis under hypoxic conditions

**DOI:** 10.3389/fcell.2023.1161541

**Published:** 2023-06-01

**Authors:** Zhan Gao, Zhicai Li, Xiaowei Li, Jun Xiao, Cuiying Li

**Affiliations:** ^1^ Department of Blood Transfusion, Air Force Medical Center, Beijing, China; ^2^ The Fifth School of Clinical Medicine, Anhui Medical University, Hefei, Anhui, China

**Keywords:** erythropoiesis, EPAS1, IRS2, hypoxia, K562 cells

## Abstract

Red blood cells (RBCs) produced *in vitro* have the potential to alleviate the worldwide demand for blood transfusion. Hematopoietic cell differentiation and proliferation are triggered by numerous cellular physiological processes, including low oxygen concentration (<5%). In addition, hypoxia inducible factor 2α (HIF-2α) and insulin receptor substrate 2 (IRS2) were found to be involved in the progression of erythroid differentiation. However, the function of the HIF-2α-IRS2 axis in the progression of erythropoiesis is not yet fully understood. Therefore, we used an *in vitro* model of erythropoiesis generated from K562 cells transduced with shEPAS1 at 5% O_2_ in the presence or absence of the IRS2 inhibitor NT157. We observed that erythroid differentiation was accelerated in K562 cells by hypoxia. Conversely, knockdown of EPAS1 expression reduced IRS2 expression and erythroid differentiation. Intriguingly, inhibition of IRS2 could impair the progression of hypoxia-induced erythropoiesis without affecting EPAS1 expression. These findings indicated that the EPAS1-IRS2 axis may be a crucial pathway that regulates erythropoiesis and that drugs targeting this pathway may become promising agents for promoting erythroid differentiation.

## 1 Introduction

Hypoxia affects a variety of cellular physiological processes, including energy metabolism, autophagy, cell motility, angiogenesis and erythropoiesis (Yu et al., 2022). Hypoxia-inducible factors (HIFs) were originally discovered in studies of hypoxia-induced erythropoiesis. HIFs bind to the gene encoding erythropoietin (EPO) and activate its transcription to promote erythropoiesis (Lappin and Lee, 2019). HIF is a heterodimer consisting of an alpha subunit that regulates activity and function and a constitutively expressed beta subunit. Under normoxic conditions, the HIF-α oxygen-dependent degradation domain is hydroxylated by proline hydroxylase and binds to the tumor suppressor protein VHL for ubiquitination and proteasomal degradation. However, this response is suppressed under hypoxic conditions, which leads to the stabilization of the HIF structure, and heterodimers of HIFs can activate the transcription of hundreds of target genes to regulate the adaptive response to hypoxia (Suzuki et al., 2017).

HIF-α homologous sequences include HIF-1α, HIF-2α and HIF-3α. In the hypoxic bone marrow environment, HIF-1α regulates the levels of p21 and p27, which inhibit S-phase cyclin-dependent kinases (CDKs) and mediate cell cycle arrest in hematopoietic stem cells (Jakubison et al., 2022). HIF-2α is encoded by endothelial PAS1 protein (endothelia pas domain protein 1, EPAS1), which regulates the gene expression of angiogenic growth factors, erythropoietin, VEGF and EPO (Gaspersic et al., 2021; Kristan et al., 2021). With the development of metabolic research techniques, researchers have focused on how metabolic alterations in hypoxic environments affect cell development and differentiation (Harris et al., 2013; Mylonis et al., 2019). Among the different metabolic processes, glucose metabolism influences the development and number of HSCs *in vivo* in addition to serving as the primary source of cellular energy. Transient increases in physiological glucose levels in zebrafish have dose-dependent effects on HSC development, including increased runx1 expression and the establishment of hematopoietic clusters around the aorta, gonad, and mesonephros (Harris et al., 2013). However, during glucose metabolism, insulin receptor/insulin receptor substrate, which is the most significant molecule associated with insulin signal transmission, sends upstream insulin signals to all levels and maintains intracellular glucose homeostasis, which impacts cell development and function (Thirone et al., 2006). Some studies have shown that hypoxia increases HIF expression in liver cells, and HIF-2α but not HIF-1α further upregulates the synthesis of insulin receptor substrate 2 (IRS2) in liver tissue and affects glucose metabolism (Wei et al., 2013; Riopel et al., 2020). During hematopoiesis, IRS2 regulates the metabolism and apoptosis of hematopoietic cells, and its expression gradually increases during CD34^+^ cell differentiation into erythroid cells and changes more significantly after the erythroid progenitor stage (Machado-Neto et al., 2018). However, the specific molecular mechanism by which hypoxia affects erythroid differentiation remains unclear.

Therefore, in this study, the acute myeloid leukemia-like cell line K562 was used as the research object to explore the effect of EPAS1 on the erythroid differentiation of K562 cells and the changes in the expression of IRS2 to clarify the adaptative mechanism of high-altitude hypoxia and the prevention of hypoxia-related blood diseases and provide new ideas for treatment.

## 2 Materials and methods

### 2.1 K562 cell culture and erythroid differentiation

K562 cells were cultured in RPMI-1640 medium (Sigma‒Aldrich, United States) containing 10% fetal bovine serum (FBS, Sigma‒Aldrich, United States) and 1% penicillin and streptomycin (Sigma‒Aldrich, United States). K562 cells were differentiated by erythroid induction at an initial concentration of 1 × 10^5^/mL cells. A final concentration of 40 μM hemin (Solarbio, China) and 0.1 ng/mL cytarabine (Solarbio, China) were added to the medium to induce erythroid differentiation, and the cells were placed at 37°C under hypoxic (5% CO_2_, 5% O_2_), CoCl (200 μM) and normoxic (5% CO_2_, 20% O_2_) conditions for 5 days.

### 2.2 Construction of a cell line with stable knockdown of EPAS1

There Lentiviral vectors targeting EPAS1 were produced by Hanbio Co., Ltd. (Shanghai, China) the target sequences were listed in Supplementary Table S1. After screening the interference efficiency of lentivirus, K562 cells were infected with the best sh-EPAS1 lentivirus at an MOI of 30. During viral transfection, 6 μg/mL polybrene was added to enhance lentiviral infection. To achieve stable knockdown cell lines, K562 cells were incubated in selection medium containing 2 μg/mL puromycin (Invitrogen, Carlsbad, CA, United States) beginning 48 h after infection.

### 2.3 Quantitative real-time PCR

Total RNA was extracted using TRIzol reagent (Invitrogen, United States), and the concentration and purity of the RNA samples were determined with a NanoDrop spectrophotometer (Thermo Fisher Scientific, United States). Five hundred nanograms of total RNA was transcribed into cDNA by using ReverTra Ace Master Mix with gDNA Remover (TOYOBO, Japan). qRT‒PCR was performed by using SYBR qPCR Mix (TOYOBO, Japan) on a Bio-Rad CFX96 instrument, and the primers are listed in Supplementary Table S2. The thermocycler program was set as follows: predenaturation at 95°C for 3 min, denaturation at 95°C for 20 s, annealing at 58°C for 20 s, and extension at 72°C for 20 s, for a total of 40 cycles. Melting curve analysis was performed after each run to evaluate the quality of the qRT‒PCR products.

### 2.4 Cell proliferation assay

Cells were collected and seeded into 96-well plates at 1 × 10^4^/mL cells per well. At 0, 24, 48, 72, 96, and 120 h, 10 μL of CCK8 (Glpbio, China) reagent was added to each well and incubated for 2 h in a 37°C incubator. The OD values were measured at a wavelength of 450 nm by a microplate reader (Bio-Rad, CA, United States).

### 2.5 Benzidine staining

Cells were collected by centrifugation at 1,200 rpm for 5 min, and 5,000 cells were stained with a benzidine staining kit (Solarbio, China). The positive rate of benzidine staining in 200 cells was counted under a high magnification (×40) microscope.

### 2.6 Flow cytometry

The cells were washed and resuspended in cell stain buffer (BioLegend, United States). Antibodies against CD235a-APC and CD71-PE (BioLegend, United States) and the appropriate isotype controls were used according to the manufacturer’s instructions. The gating strategy was based on the isotype controls, and analyses were performed using FlowJo software (Version 10, TreeStar).

### 2.7 Western blot analysis

Total cells were lysed in lysis buffer (Cwbio, Beijing) supplemented with protease inhibitors (Roche Mannheim, Germany) and then centrifuged at 13,000 × g for 10 min at 4°C, after which the supernatant was denatured at 100°C for 5 min with Laemmli buffer. Thirty microliters of whole protein samples were separated on 12% sodium dodecyl sulfate polymerase gel (SDS‒PAGE) and transferred to polyvinylidene fluoride (PVDF) membranes (Millipore, United States). After being blocked with 5% (*w*/*v*) skim milk, the membranes were incubated with primary antibodies overnight at 4°C, and the bands were analyzed with a Bio-Rad ChemiDoc XRS imaging system.

### 2.8 Strategy for bioinformatics analysis

Gene expression profiles of erythroid differentiation in K562 cells under hypoxic culture conditions were taken from the GEO database (GSE199778). The bioinformatics platform Sangerbox Tools (Shen et al., 2022) was used to determine the differential gene expression and for figure production. The raw *p* values were adjusted by Benjamini and Hochberg multiple comparison methods to control the false discovery rate (FDR). If the corrected *p*-value was less than 0.05, the corresponding mRNAs were statistically significant and subjected to Kyoto Encyclopedia of Genes and Genomes (KEGG) pathway analysis using DAVID Bioinformatics Resources (version 6.8).

### 2.9 Statistics

The SPSS statistical program V.26 (SPSS Inc., Chicago, IL, United States) was used to conduct data analyses, and all values are expressed as the means ± standard deviations (SD). Significant differences were evaluated by two-tailed Student’s t-test when two groups were compared and one-way analysis of variance (ANOVA) followed by the LSD test when performing multiple comparisons between groups. A statistically significant difference was indicated by a *p*-value less than 0.05.

## 3 Results

### 3.1 Hypoxia enhances K562 cell differentiation into erythroid cells

**FIGURE 1:**
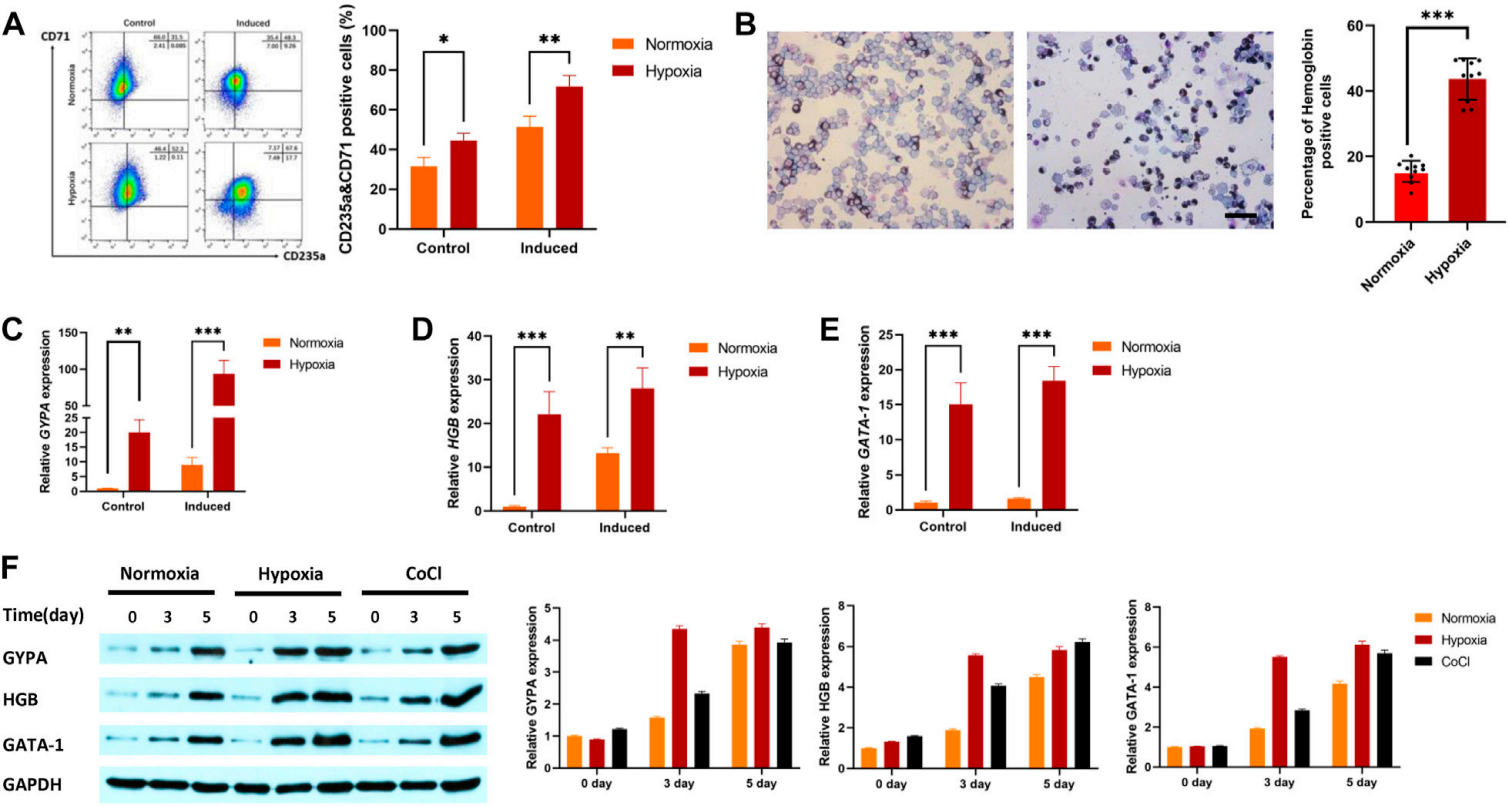
Hypoxia promoted erythroid differentiation in K562 cells. **(A)** K562 cells were treated with or without hemin (40 μM) and cytarabine (0.1 ng/mL) for 5 days under hypoxia. CD71 and CD235 were detected by flow cytometry. **(B)** To determine whether cells with hemoglobin had been produced, the cells were stained with benzidine (40×, bar = 50 μm). A total of 1,000 cells were counted in various visual fields on each of ten different benzidine-stained slides, and the quantity of hemoglobin-positive cells was noted. The average value was used to calculate the percentage of cells that were hemoglobin-positive. **(C–F)** Hypoxia promoted the expression of GYPA, HBG, and GATA-1 mRNA and protein in K562 cells. Cells were exposed to 5% O_2_ or CoCl (200 μM) for 5 days. GYPA, HBG and GATA-1 transcripts were measured by qRT‒PCR **(C–E)**. GYPA, HBG, and GATA-1 protein expression levels were detected by Western blotting using GAPDH to calibrate sample loading **(F)**. The data are presented as the mean ± SD of three independent experiments. *p < 0.05, **p < 0.01 and ***p < 0.001 for treated cells *versus* normoxic cells by Student’s *t* test.

The progressive increase in the percentage of CD235a and CD71 double-positive cells is a sign that K562 cells are developing into erythroid cells. The results showed that the percentage of positive cells for CD235a and CD71 under hypoxic conditions was higher than that under normoxic conditions with or without erythroid induction (Figure 1A). Benzidine staining was further used to detect changes in hemoglobin levels during erythroid induction after hypoxic stimulation. The results indicated that the percentage of benzidine-positive cells after hypoxia exposure (43.67%) was higher than that after normoxia exposure (15.44%, Figure 1B). qRT‒PCR was used to detect the gene expression of IRS2, EPAS1, *γ*-hemoglobin (HGB) and CD235a in K562 cells. The results indicated that the gene expression of glycophorin-A (GYPA, Figure 1C), hemoglobin (HGB, Figure 1D) and GATA-1 (Figure 1E) in K562 cells was significantly increased. Furthermore, the expression of these three proteins increased after 3 days or 5 days of hypoxia exposure (in 5% O_2_ culture or CoCl treatment, Figure 1F), which was consistent with the qRT‒PCR and benzidine staining results.

### 3.2 EPAS1 acts as the key factor in K562 erythroid differentiation

**FIGURE 2:**
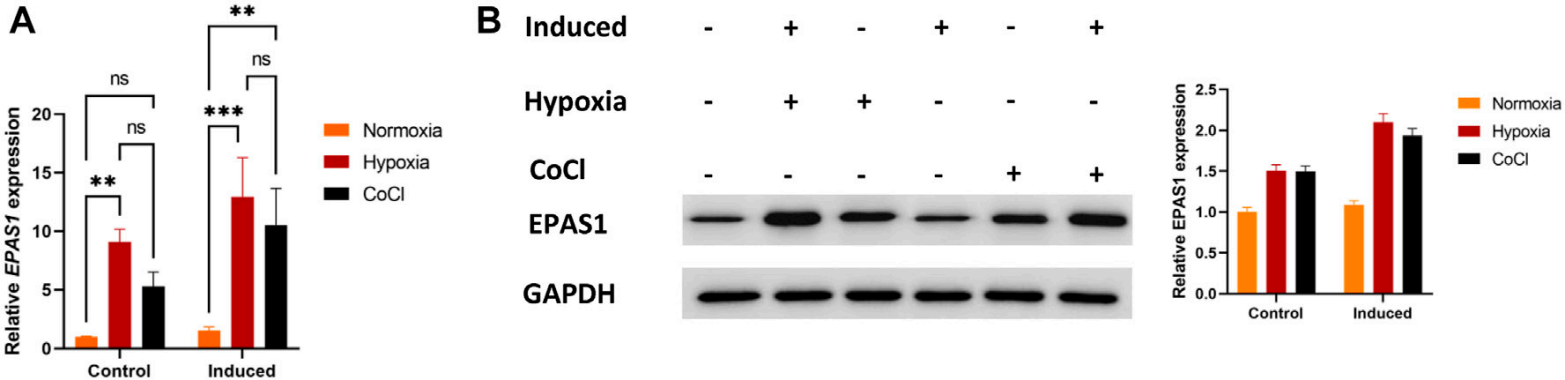
Hypoxia promoted EPAS1 gene expression in K562 cells. After the cells were treated with 5% O_2_ or CoCl (200 μM) for 5 days, EPAS1 gene and protein expression were measured by qRT‒PCR **(A)** and Western blotting **(B)**. The data are presented as the mean ± SD of three independent experiments. **p < 0.01 and ***p < 0.001 for different treated cells by LSD analysis.

First, we evaluated whether EPAS1 participated in K562 erythroid differentiation under hypoxic stimulation. The expression of EPAS1 mRNA (Figure 2A) and protein (Figure 2B) was increased under low-O_2_-induced (5% O_2_) hypoxia or CoCl-induced hypoxic stimulation.

**FIGURE 3:**
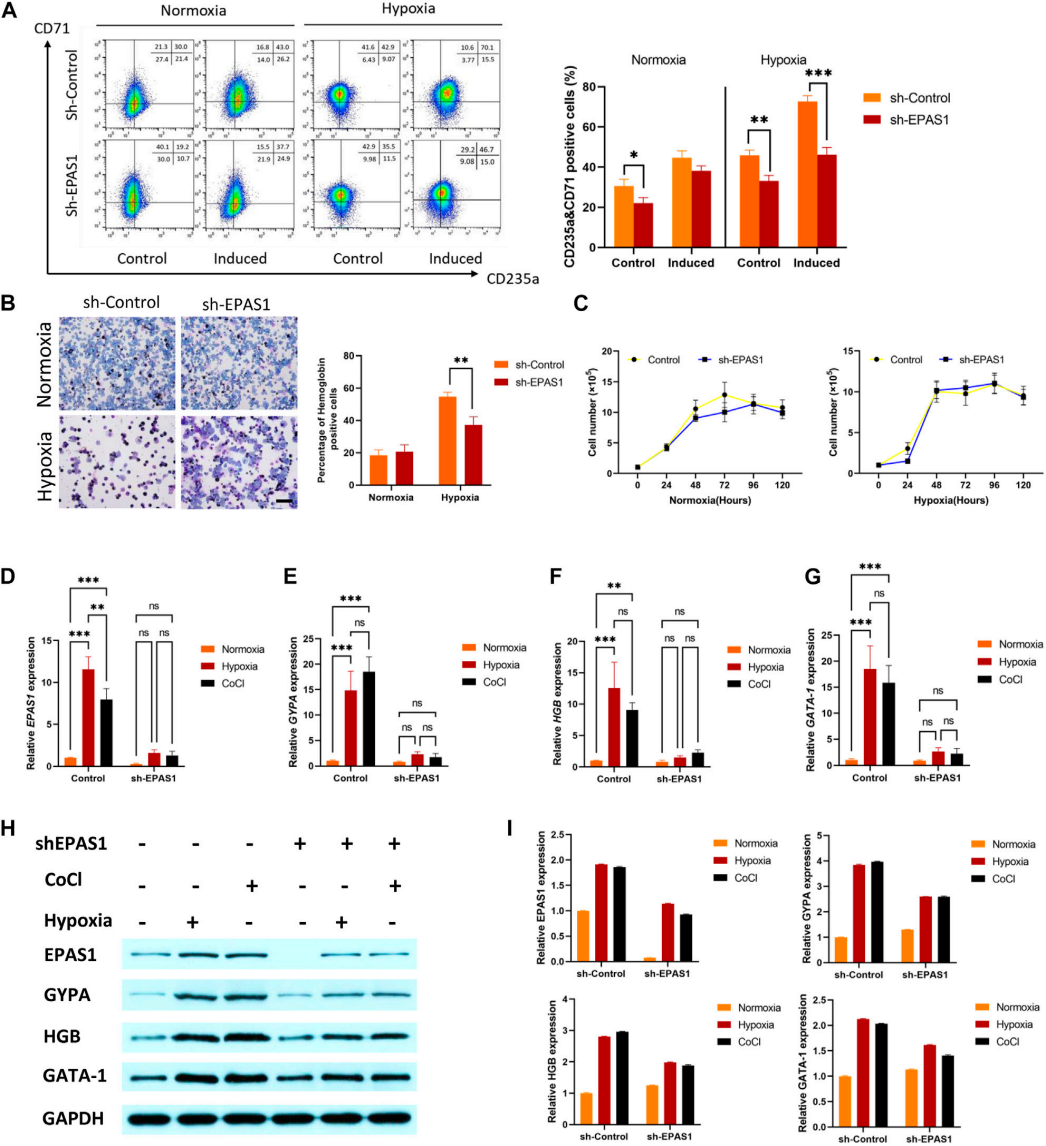
EPAS1 was a crucial factor in hypoxia-induced erythropoiesis in K562 cells. After the EPAS1 gene was knocked down in K562 cells, the cells were cultured under hypoxic conditions for 5 days. **(A)** The percentage of CD71-and CD235a-positive cells was determined by flow cytometry. **(B)** On each of ten distinct benzidine-stained slides, a total of 1,000 cells were counted in various visual areas, and the number of hemoglobin-positive cells was recorded (40×, bar = 50 μm). The average values of the proportion of hemoglobin-positive cells are shown in 5-day culture. **(C)** Wild-type K562 cells and EPAS1-knockdown cells were seeded in 96 culture dishes. The OD values produced by the CCK-8 test were used to examine cell proliferation at 0, 24, 48, 72, 96, and 120 h **(D–G)** The gene expression of EPAS1, GYPA, HGB and GATA-1 was measured by qRT‒PCR. **(H–I)** The protein expression levels of EPAS1, GYPA, HGB and GATA-1 were evaluated in K562 cells determined on Day 5 of culture, as well as cells with shEPAS1 under hypoxic or CoCl (200 μM) treatment conditions, by Western blotting. The data are presented as the mean ± SD of three independent experiments. *p < 0.05, **p < 0.01 and ***p < 0.001 for different treated cells by Student’s *t* test or LSD analysis.

To determine the putative role of EPAS1 in erythropoiesis under hypoxic conditions, K562 cells were infected with a lentivirus carrying a small hairpin RNA against EPAS1 (sh-EPAS1). The cells were transduced for 5 days and then cultured for an additional 5 days in the presence of hemin (40 μM) and cytarabine (0.1 ng/mL). The flow cytometry and benzidine staining results indicated that incubation in hypoxic conditions alone increased the percentages of CD235a+/CD71+ cells (Figure 3A) and hemoglobin-positive cells (Figure 3B), while EPAS1 silencing (sh-EPAS1) reduced K562 erythroid differentiation (Figures 3A, B). We further considered whether this phenomenon was caused by a decrease in K562 cell counts after EPAS1 silencing. Interestingly, cell expansion in the presence or absence of hypoxic culture conditions was rarely affected (Figure 3C). Furthermore, erythropoiesis-related gene expression indicated that EPAS1 gene expression was markedly increased under hypoxia, while EPAS1 silencing inhibited the expression of these genes (Figures 3D–G). Additionally, the protein expression results were consistent with the previous results (Figures 3H, I). And the main results were repeated in the human erythroleukemia cell line (HEL, Supplementary Figure S2). These results suggest that EPAS1 may be a key factor in the erythrogenic differentiation of K562 cells under hypoxia.

### 3.3 HIF-2α mediates K562 erythropoiesis through IRS2

**FIGURE 4:**
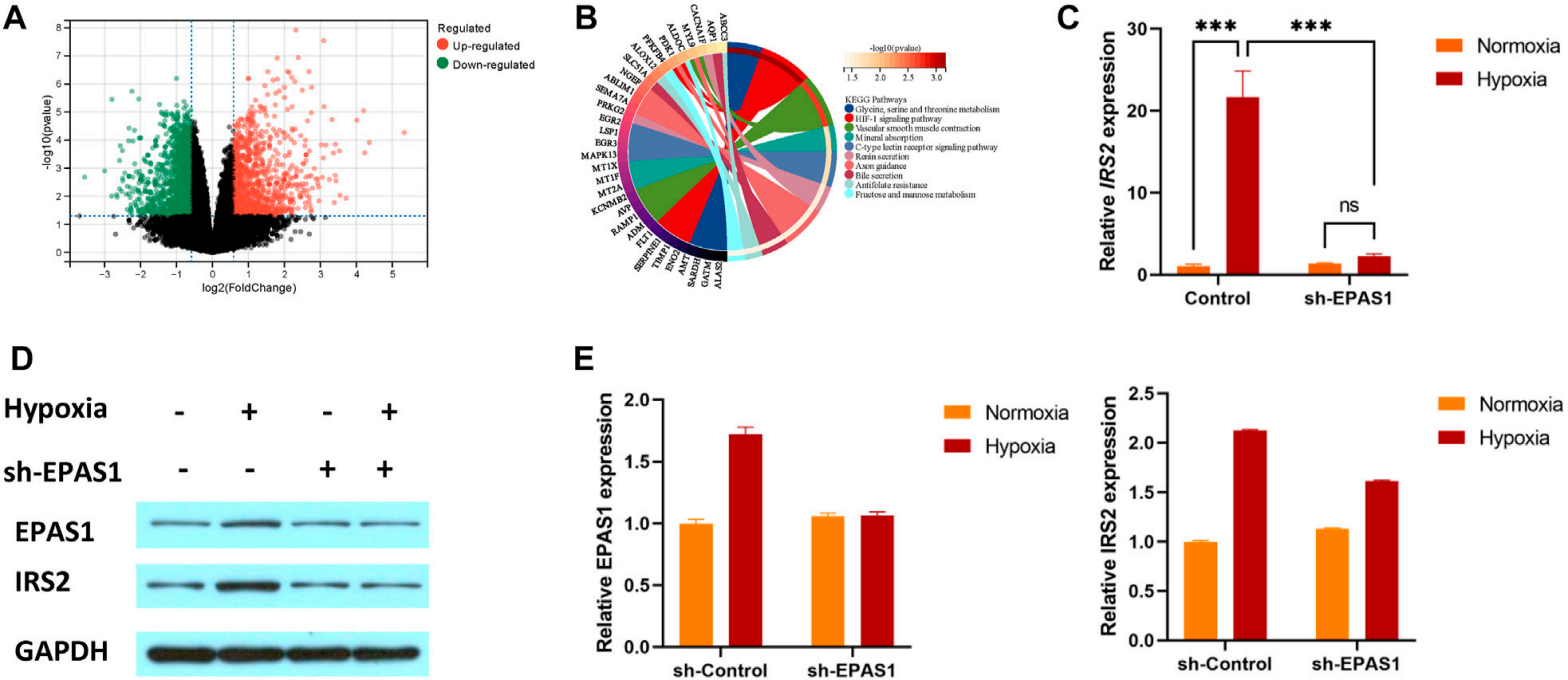
IRS2 may be a key factor in EPAS1-mediated regulation of erythroid differentiation in K562 cells under hypoxic conditions. **(A)** Volcano plot of differentially expressed genes in K562 cells under hypoxia. The red dot represents upregulated genes, and the green dot represents downregulated genes. **(B)** The significantly enriched KEGG pathways of target genes. **(C–E)** The gene and protein expression of IRS2 in K562 cells in the presence or absence of sh-EPAS1 under hypoxic conditions was measured by qRT‒PCR and Western blotting. The data are presented as the mean ± SD of three independent experiments. ***p < 0.001 for different treated cells by LSD analysis.

To understand the specific mechanism of erythroid differentiation in K562 cells under hypoxic culture conditions, we further analysed the GSE199778 dataset for K562 hypoxic culture in the GEO database. The results showed that 299 genes were upregulated and 195 were downregulated (Figure 4A). KEGG analysis showed that multiple genes were involved in carbohydrate and amino acid metabolism (Figure 4B). We verified that the IRS2 gene as involved in glucose metabolism and found that the expression of the IRS2 gene and protein was significantly increased in K562 cells under hypoxic conditions. Moreover, the gene (Figure 4C) and protein expression (Figures 4D, E) levels of IRS2 were significantly inhibited after interference with EPAS1 expression. These results suggest that EPAS1-mediated erythrogenic differentiation of K562 cells under hypoxia may be mediated by IRS2.

### 3.4 The IRS2 acts as a key factor during the differentiation of hypoxia-enhanced erythropoiesis

**FIGURE 5:**
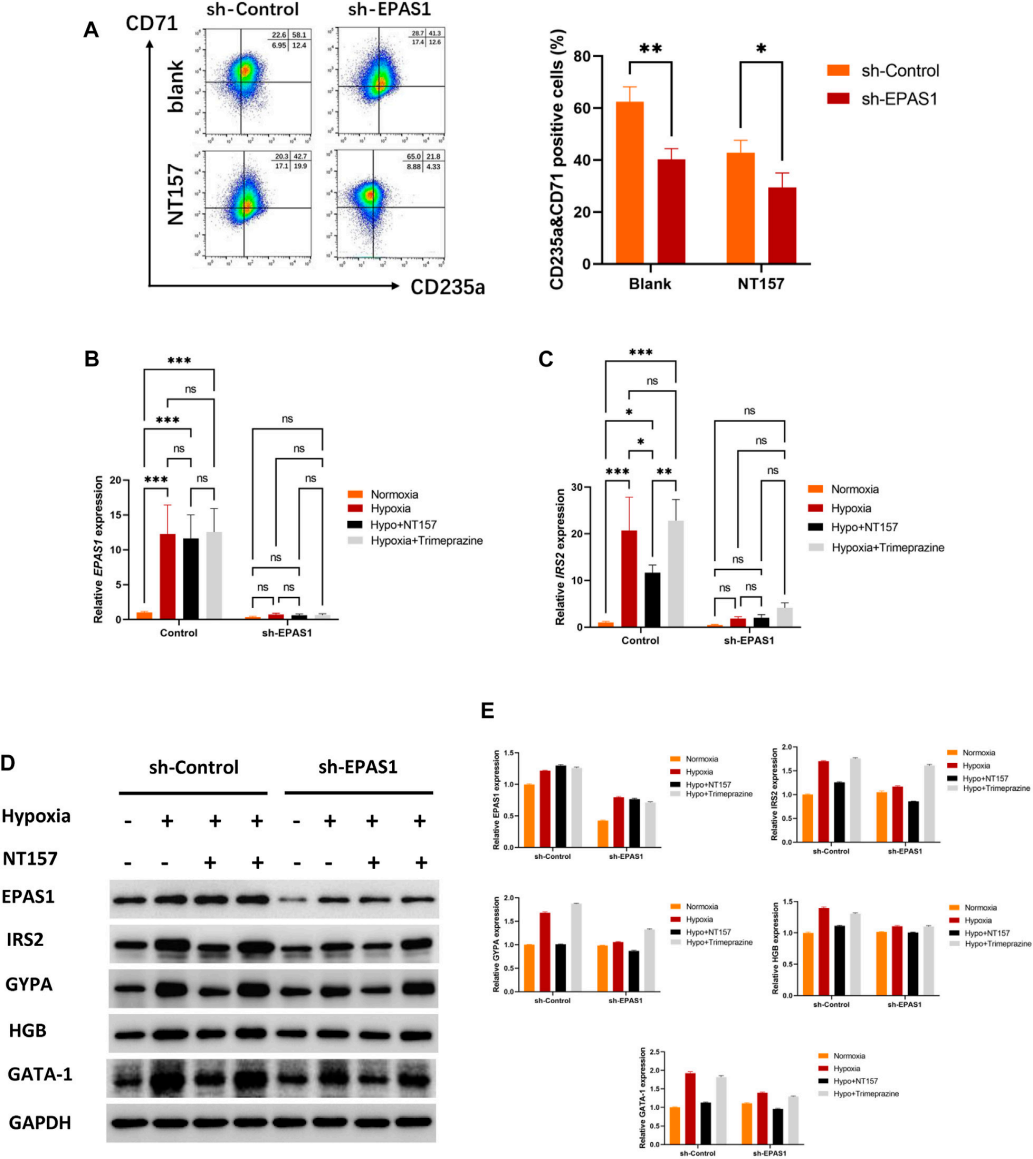
The EPAS1-IRS2 axis is one of the key regulators of hypoxia-induced erythroid differentiation. NT157 and Trimeprazine, the inhibitor and agonists of IRS2, were used to verify the effect of EPAS1-IRS2 on erythroid differentiation in K562 cells and EPAS1-knockdown cells under hypoxic conditions. **(A)** The percentage of CD71-and CD235a-positive cells was determined by flow cytometry. The bar charts show the average percentage of CD71+/CD235a+ cells in 5-day cultures. **(B–C)** IRS2 and EPAS1 gene expression was measured by qRT‒PCR. **(D–E)** EPAS1, IRS2, GYPA, HBG and GATA-1 protein expression levels were detected by Western blotting using GAPDH to calibrate sample loading. The data are presented as the mean ± SD of three independent experiments. *p < 0.05, **p < 0.01 and ***p < 0.001 for different treated cells by Student’s *t* test or LSD analysis.

To further clarify the role of IRS2 in the erythroid differentiation of K562 cells, NT157 and Trimeprazine, the inhibitor and agonists of IRS2, were added to the cell culture medium. The flow cytometry results showed that K562 cells had high levels of erythroid differentiation when exposed to hypoxia, while K562 erythroid differentiation could be significantly inhibited when cells were transfected with the sh-EPAS1 lentivirus or treated with NT157 (Figure 5A). Moreover, interference with EPAS1 and inhibition of IRS2 at the same time further reduced erythroid differentiation (Figure 5A). The specific effect of IRS2 was verified at the gene (Figures 5B, C) and protein levels (Figures 5D, E). The results showed that NT157 could significantly inhibit erythroid differentiation induced by hypoxia, and erythroid differentiation was further hindered by interfering with EPAS1 in the presence of NT157. However, Trimeprazine saved the hindered erythroid differentiation caused by interfering with EPSA1 by activating IRS2. These results suggested that the EPAS1-IRS2 axis plays an important role in the erythroid differentiation of K562 cells induced by hypoxia**.**

## 4 Discussion

The synthesis of hemoglobin and the creation of blood cells have been shown to be intimately related to hypoxia, as demonstrated by the bone marrow hypoxia niche during the hematopoietic process and the greatly increased hematopoietic capacity in the hypoxic environment of the body (Befani and Liakos, 2018; Wielockx et al., 2019). In the present study, we used K562 cells, which is a common model to study erythroid differentiation that is rich in glycophorin A and can synthesize embryonic and fetal hemoglobin (Uchida et al., 2018). The results showed that the percentage of cells that were positive for CD235a and CD71 increased compared with the normoxia group, and the difference was more obvious after induction. We examined whether hypoxia alone could promote erythroid differentiation in the absence of inducers and whether hypoxia could further boost the efficacy of erythroid induction when it occurred under induced conditions. This change is quite important *in vivo*, and it might be connected to the alterations in the levels at which chemicals are metabolized in cells that have been activated by hypoxia. The related factors or pathways are activated by an increase or reduction in these metabolites, which encourages the differentiation of cells that can produce enough red blood cells to maintain the oxygen supply to tissue cells. However, the precise process by which hypoxia alters the cell phenotype or the direction of differentiation is still unknown.

During erythropoiesis, HIF-2α seems to have a more prominent role than HIF-1α (Kapitsinou et al., 2010; Gkotinakou et al., 2021). The results of the present study also showed that both physical hypoxia (5%O_2_) and chemical hypoxia (CoCl stimulation) can promote the expression of EPAS1 in K562 cells. It has been demonstrated that knockout of HIF-2α decreases bone volume in mice, blocks erythroid differentiation in hematopoietic cells, downregulates the expression of genes related to iron transport and release, and reduces hemoglobin synthesis (Krock et al., 2015). Furthermore, a similar phenomenon was observed in HIF-knockout human embryonic stem cells, and the effect of HIF-2α knockout on the hematopoietic system was more pronounced than that of HIF-1α (Xie et al., 2019). Further evidence that HIF-2α knockdown can attenuate erythroid differentiation in K562 cells under hypoxic conditions but does not alter cell proliferation was found in this study, indicating that HIF-2α may be a key factor in the erythrogenic differentiation of K562 cells under hypoxia. However, these effects were weak in normoxia stimulation. The possible reason for this phenomenon is that under normal oxygen conditions, HIF-2α will be hydroxylated by proline hydroxylase (PHD), causing HIF-2α binding to von Hipplel-Lindau (VHL), which recruits E3 ubiquitinase binding complex and ultimately causes HIF-2α ubiquitination degradation (Scheuermann et al., 2013). However, under hypoxia conditions, the activity of PHD is significantly decreased, resulting in the inhibition of HIF-2α hydroxylation, and HIF-2α expression is increased to regulate the expression of target genes (Kaelin and Ratcliffe, 2008). Therefore, the interference effect on cells differentiation by treated with sh-EPAS1 is more obvious. Furthermore, according to genotyping studies of other populations, the abundant EPAS1 gene variant in the Tibetan population is one of the reasons for the low expression level of this gene, which is conducive to maintaining normal hemoglobin and red blood cell levels (Peng et al., 2017).

Studies have shown that stable knockout of hepatocyte PHD can specifically stabilize HIF-2α and enhance the expression of IRS2, which showed that HIF-2α was involved in hepatocytes and insulin signalling (Wei et al., 2013), but the role of HIF-2α in erythroid differentiation cell line models and IRS2 expression is still unclear. IRS2, which is an adaptor protein, can be recruited and activated by insulin-like growth factor receptor, erythropoietin receptor and thrombopoietin receptor and activate various signalling pathways, such as PI3K/AKT/mTOR and MAPK, to regulate cell growth, metabolism and proliferation. Differentiation (de Melo Campos et al., 2016). Similarly, our findings indicated that multiple genes were involved in carbohydrate and amino acid metabolism in the presence of hypoxia, indicating that genes involved in metabolism, such IRS2, may be crucial for K562 cells to differentiate into erythroid cells under these conditions. The degree of IRS2 deficiency was inversely correlated with the reduction in hematopoietic cells in patients with myelodysplastic syndrome (Machado-Neto et al., 2018), demonstrating the importance of an IRS2 level that is within the normal range for healthy hematopoiesis. The results of the present study showed that hypoxic stimulation could promote the expression of IRS2, but this effect was significantly inhibited by EPAS1 knockdown, suggesting that the EPAS1-IRS2 axis may be a key pathway affecting erythropoiesis in K562 cells under hypoxia.

To understand the effect of the EPAS1-IRS2 axis on erythropoiesis, we used NT157 to block IRS2 and examined its impact on erythroid differentiation in K562 cells. We discovered that after inhibiting IRS2, the effect of hypoxia on EPAS1 remained unchanged, but the genes and proteins related to erythroid differentiation were inhibited, further indicating the stimulatory effect on the EPAS1-IRS2 axis under hypoxia. However, there are still some limitations in this study. First, although we simulated the *in vivo* hypoxic environment using *in vitro* 5% O_2_ stimulation and obtained some crucial experimental data, it is still unclear whether these results would necessarily apply *in vivo*. Therefore, additional *in vivo* experiments are required to verify the regulatory mechanisms in the present study. Second, K562 and HEL cells, which are acute myeloid leukemia-like cell line and human erythroleukemia cell line, and hematopoietic stem cells have certain similarities regarding erythropoietic development, but there are still some differences between the two cell types (Deezagi, 2014; Song et al., 2020). Finally, the function of the EPAS1-IRS2 axis in K562 cell erythroid development has been researched here, but its precise molecular mechanism has not been completely investigated. Previous research has demonstrated that EPO mediates CFU-E expansion through the regulation of IRS2 expression by JAK2/STAT5 (Hsieh et al., 2022) and HIF-2α regulates IRS2 through Srebp1c-dependent and–independent mechanisms (Wei et al., 2013), which has a significant effect on directions for ongoing investigations.

In conclusion, hypoxia can upregulate the expression levels of EPAS1 and IRS2 in the erythroid differentiation model of K562 cells, and inhibiting EPAS1 and IRS2 could attenuate hypoxia-induced erythropoiesis. This study preliminarily explored the role of EPAS1-IRS2 in hypoxia-induced erythroid differentiation, which could provide a new theoretical basis for high-altitude hypoxia adaptation and hypoxia-related blood diseases.

## 5 Additional requirements

**Institutional Review Board Statement** The study was conducted according to the guidelines of the Declaration of Helsinki and approved by the Institutional Review Board of Air Force Medical Center (2021-134-YJ01).

## Data Availability

The original contributions presented in the study are included in the article/Supplementary Material, further inquiries can be directed to the corresponding authors.
