# Supplementary material for: Regulation of erythroid differentiation in K562 cells by the EPAS1-IRS2 axis under hypoxic conditions
Source: Front Cell Dev Biol. 2023 Jun 1;11:1161541. doi: 10.3389/fcell.2023.1161541 (PMC10267359; doi:10.3389/fcell.2023.1161541)
Supplement: Supplementary file 1 [file Table1.DOCX]

Supplementary Material

Regulation of Erythroid Differentiation in K562 Cells by the EPAS1-IRS2 Axis under Hypoxic Conditions

Zhan Gao1, Zhicai Li 2, Xiaowei Li 1, Jun Xiao 1*, Cuiying Li 1,2*

*** Correspondence:** Corresponding Author: ammsxj@fmmu.edu.cn; lcy2013@fmmu.edu.cn

# Supplementary Table1 shRNA target sequences

| shRNA name | Target sequences （5′→3′） |
| --- | --- |
| shControl | TTCTCCGAACGTGTCACGTAA |
| shEPAS1-1 | CAGGTGGAGCTAACAGGACATAGTA |
| shEPAS1-2 | GAACTTCGAGGAGTCCTCAGCCTAT |
| shEPAS1-3 | CCCTCTCCAACAAGCTGAAGCTGAA |

# Supplementary Table2 qRT-PCR primer sequences

| Primers name | sequences （5′→3′） |
| --- | --- |
| EPAS1-F | GCGACCATGAGGAGATTCGT |
| EPASI-R | GACCGTGCACTTCATCCTCA |
| IRS2-F | CTCACCCTGTAGTGCCTTCG |
| IRS2-R | GCAGTGGGTACTCGTGCAT |
| GAPDH-F | ATTCCATGGCACCGTCAAGG |
| GAPDH-R | TGGACTCCACGACGTACTCA |
| GATA1-F | AAACGGGCAGGTACTCAGTG |
| GATA1-R | CGGTTCACCTGGTGTAGCTT |
| GYPA-F | TGATACGCACAAACGGGACA |
| GYPA-R | ACCAGCCATCACCCCAAAAA |
| HGB-F | GAGAAACCCTGGGAAGGCTC |
| HGB-R | TGTGCCTTGACTTTGGGGTT |
