# Supplementary material for: Regulation of erythroid differentiation in K562 cells by the EPAS1-IRS2 axis under hypoxic conditions
Source: Front Cell Dev Biol. 2023 Jun 1;11:1161541. doi: 10.3389/fcell.2023.1161541 (PMC10267359; doi:10.3389/fcell.2023.1161541)

Supplementary figure1

shEPAS1-1: CAGGTGGAGCTAACAGGACATAGTA

shEPAS1-2: GAACTTCGAGGAGTCCTCAGCCTAT

shEPAS1-3: CCCTCTCCAACAAGCTGAAGCTGAA

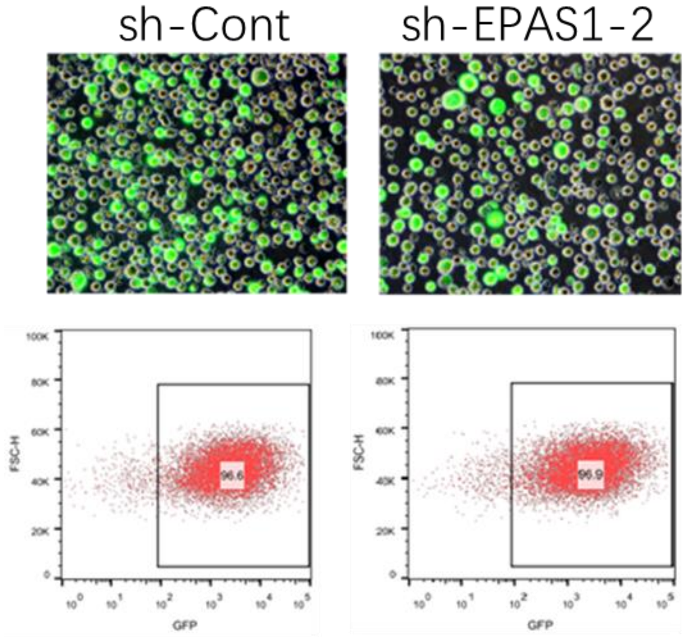

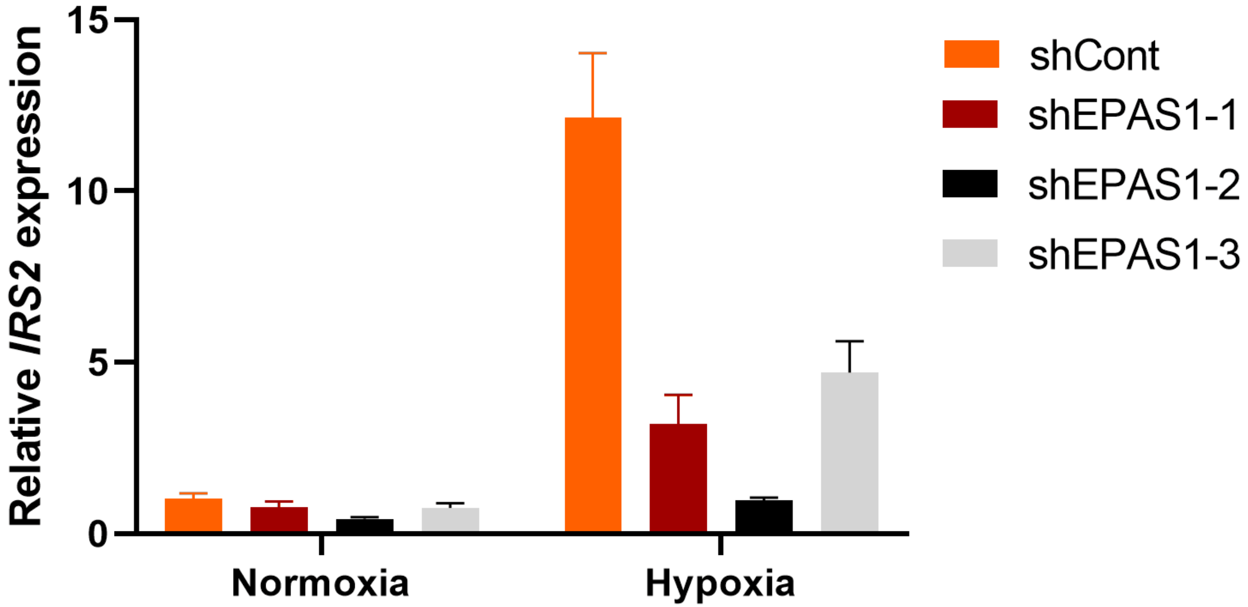

Supplementary figure2

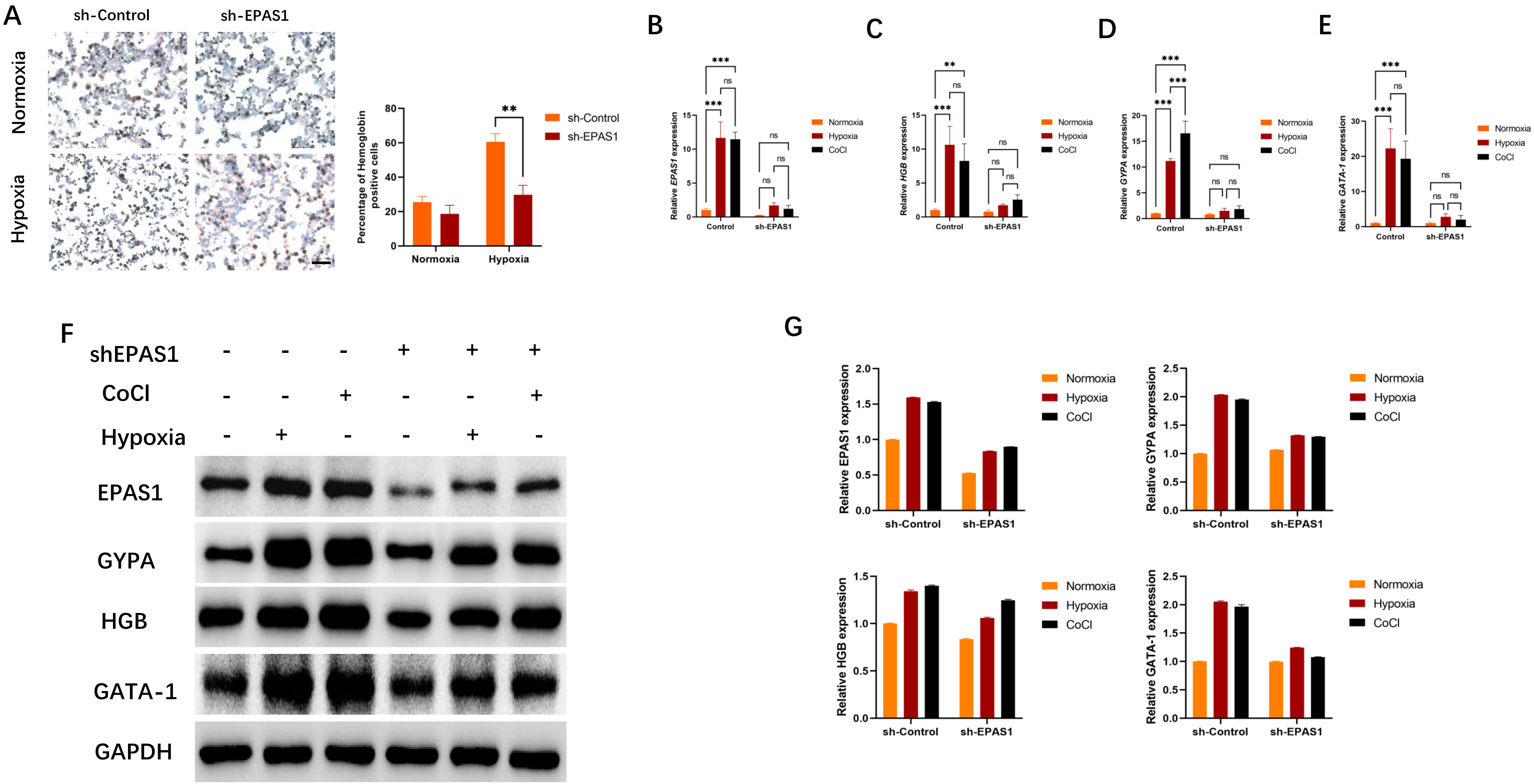

Supplement: Supplementary file 2 [file DataSheet1.PDF]
